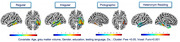# Deciphering Dyslexia Phenotypes in Chinese Language Users with Primary Progressive Aphasia

**DOI:** 10.1002/alz70857_106949

**Published:** 2025-12-26

**Authors:** Boon Lead Tee, YuChen Chuang, Lung‐Tat Chan, Lorinda Li‐Ying Kwan Chen, Ta‐Fu Chen, Raymond Y. Lo, Joshua Tsoh, Adrian Wong, Chien Jung Lu, Yu Sun, Pei‐Ning Wang, YiChen Lee, Isabel Elaine Allen, Maria Luisa Mandelli, Yu‐Ruei Lin, Fangda Leng, Maria Luisa Gorno Tempini

**Affiliations:** ^1^ Memory and Aging Center, Department of Neurology, University of California San Francisco, San Francisco, CA, USA; ^2^ Global Brain Health Institute, University of California, San Francisco, San Francisco, CA, USA; ^3^ University of California, San Francisco, San Francisco, CA, USA; ^4^ Division of General Neurology, Department of Neurological Institute, Taipei Veterans General Hospital, Taipei, Taipei, Taiwan; ^5^ Department of Medicine, Queen Elizabeth Hospital, Hong Kong, Hong Kong; ^6^ Department of Special Education and Counselling, The Education University of Hong Kong, Hong Kong, Hong Kong; ^7^ Department of Neurology, National Taiwan University Hospital, Taipei, Taiwan; ^8^ Center for Dementia Care, St. Mary's Hospital Taitung, Taiwan; Department of Biochemistry and Molecular Medicine, National Dong Hwa University, HuaLien, HuaLien, Taiwan; ^9^ Prince of Wales Hospital and ShaTin Hospital, Ma On Shan, New Territories, Hong Kong; ^10^ Gerald Choa Neuroscience Institute, The Chinese University of Hong Kong, Hong Kong SAR, Hong Kong; ^11^ En Chu Kong Hospital, New Taipei City, Taiwan; ^12^ Department of Neurology, En Chu Kong Hospital, Taipei, Taiwan; ^13^ Taipei Veterans General Hospital, Taipei, Taiwan; ^14^ Department of Neurology, National Taiwan University Hospital HsinChu branch, HsinChu, HsinChu, Taiwan; ^15^ Department of Epidemiology and Biostatistics, University of California, San Francisco, San Francisco, CA, USA; ^16^ Global Brain Health Institute, University of California, San Francisco, CA, USA, San Francisco, CA, USA, San Francisco, CA, USA; ^17^ Memory and Aging Center, UCSF Weill Institute forNeurosciences, University of California, San Francisco, San Francisco, CA, USA, San Francisco, CA, USA; ^18^ Memory and Aging Center, Department of Neurology, Weill Institute for Neurosciences, University of California, San Francisco, San Francisco, CA, USA

## Abstract

**Background:**

The diverse typology of languages often precipitates distinct language‐specific symptomatologies. While dyslexia and dysgrahia are included in the diagnostic criteria of Primary Progressive Aphasia (PPA), the descriptions predominantly pertain to alphabetic scripts, with a lack of insight into their manifestations in logographic systems. This study examines the dyslexia phenotypes of Chinese‐speaking PPA patients.

**Method:**

The Chinese Language Assessment for PPA (CLAP) study recruited Mandarin and Cantonese‐speaking participants [cognitively normal (CN, *n* = 68) and individuals with PPA (16 semantic variant (sv)PPA, 16 nonfluent/aggramatic variant (nfv)PPA ), 21 logopenic variant (lv)PPA] using a neurolinguistic tailored battery for Chinese languages. In the CLAP character reading test, participants are required to read 250 Chinese characters, encompassing a range of lexical types (pictographic, regular compound, and irregular compound characters), frequencies, and levels of concreteness. Participants are also tasked to read aloud pairs of compound words that include heteronyms (i.e., words with same spelling but pronounced differently; e.g., 'bow tie' versus 'bow down'). Additionally, voxel‐based morphometry was utilized to investigate the neural basis of reading performance.

**Result:**

In the character reading test, svPPA participants demonstrated significantly lower performance than other study groups, even after adjusting for age, education, and testing language (pictographic: *p* <0.001; regular compound: *p* <0.001; irregular compound characters: *p* <0.001). While over‐regularization errors (i.e., surface dyslexia) were prevalent across control and PPA groups, they were not specific to svPPA (*p* = 0.495). In contrast, on the heteronym word reading test, both svPPA and lvPPA showed significantly lower accuracy (both *p* <0.001), with over‐regularization errors occurring more frequently in the svPPA group (*p* <0.001). Across all lexical categories, character reading scores were significantly correlated with volumetric changes in the left middle and inferior temporal regions while heteronym reading accuracy were positively correlated with left temporal pole and inferior temporal areas.

**Conclusion:**

Contrary to English svPPA patients who primarily struggled with irregular word reading and frequently exhibit surface dyslexia, Chinese PPA patients showed no variant‐specific differences across lexicality, and svPPA‐specific over‐regularization phenomenon were found at the lexical (i.e., heteronym‐reading) and not sub‐lexical reading. These findings underscore that diagnostic criteria for PPA syndromes should be linguistically tailored to accommodate language topology effect.